# 17β-Estradiol Regulates miR-9-5p and miR-9-3p Stability and Function in the Aged Female Rat Brain

**DOI:** 10.3390/ncrna7030053

**Published:** 2021-08-30

**Authors:** Chun K. Kim, Megan L. Linscott, Sarah Flury, Mengjie Zhang, Mikayla L. Newby, Toni R. Pak

**Affiliations:** Department of Cell and Molecular Physiology, Stritch School of Medicine, Loyola University, Maywood, IL 60153, USA; chun9kim@gmail.com (C.K.K.); mlinscott@luc.edu (M.L.L.); sflury@luc.edu (S.F.); mzhang5@luc.edu (M.Z.); mnewby1@luc.edu (M.L.N.)

**Keywords:** miR-9-5p, miR-9-3p, brain, aging, menopause

## Abstract

Clinical studies demonstrated that the ovarian hormone 17β-estradiol (E_2_) is neuroprotective within a narrow window of time following menopause, suggesting that there is a biological switch in E_2_ action that is temporally dependent. However, the molecular mechanisms mediating this temporal switch have not been determined. Our previous studies focused on microRNAs (miRNA) as one potential molecular mediator and showed that E_2_ differentially regulated a subset of mature miRNAs which was dependent on age and the length of time following E_2_ deprivation. Notably, E_2_ significantly increased both strands of the miR-9 duplex (miR-9-5p and miR-9-3p) in the hypothalamus, raising the possibility that E_2_ could regulate miRNA stability/degradation. We tested this hypothesis using a biochemical approach to measure miRNA decay in a hypothalamic neuronal cell line and in hypothalamic brain tissue from a rat model of surgical menopause. Notably, we found that E_2_ treatment stabilized both miRNAs in neuronal cells and in the rat hypothalamus. We also used polysome profiling as a proxy for miR-9-5p and miR-9-3p function and found that E_2_ was able to shift polysome loading of the miRNAs, which repressed the translation of a predicted miR-9-3p target. Moreover, miR-9-5p and miR-9-3p transcripts appeared to occupy different fractions of the polysome profile, indicating differential subcellular. localization. Together, these studies reveal a novel role for E_2_ in modulating mature miRNA behavior, independent of its effects at regulating the primary and/or precursor form of miRNAs.

## 1. Introduction

microRNAs (miRNAs) are small non-coding RNAs that post-transcriptionally regulate up to 60% of all cellular proteins [1]. Consequently, miRNAs are poised to control dynamic signaling cascades by adjusting the absolute amount of proteins available required to accomplish multiple physiological processes, thereby allowing cells to rapidly adapt to changing internal and external stimuli. Aging can be considered a chronic stimulus that elicits a complex array of cellular changes, many of which are the direct result of reduced levels of proteins that protect the cell against age-related damage [2]. The ovarian hormone 17β-estradiol (E_2_) also regulates the amount of proteins that can contribute to cellular protection in many tissues, especially in the brain; however, this E_2_-mediated protection is lost following the menopausal transition and advanced age [3]. The “Timing Hypothesis” speculates that E_2_ is neuroprotective within a critically narrow window of time following menopause, suggesting that there is a biological switch in E_2_ action that is temporally dependent [4]. Our previously published work raised the possibility that miRNAs could underlie this temporal switch in E_2_ action in the aging female brain. For instance, miR-9-5p and miR-9-3p were differentially regulated by E_2_ in the hypothalamus and hippocampus of aged female rats [5,6]. This finding was particularly relevant due to the extensively characterized roles of miR-9-5p and miR-9-3p in maintaining healthy neuronal function through their regulation of neuronal differentiation, dendritic branching, and apoptosis [7,8,9,10,11,12]. Notably, our previous data demonstrated that the ability of E_2_ to regulate these miRNAs in aged rats was dependent on the length of time between ovarian hormone depletion (via ovariectomy) and the subsequent timing of replacement E_2_ treatment [6]. Those data further supported the notion that differential regulation of miR-9-5p and miR-9-3p could, in part, explain the disparate functional outcomes of E_2_ treatment in women closer to the menopausal transition compared to those given E_2_ many years past menopause. 

Regulation of miRNAs by E_2_ can theoretically be achieved at multiple steps in the miRNA biogenesis pathway. Briefly, miRNA biogenesis is initiated by the transcription of a long primary transcript (pri-miRNA) which is subsequently cleaved by the RNase III enzyme (DROSHA) to form the precursor (pre-miRNA); after nuclear export, pre-miRNA is cleaved by another RNase III enzyme (DICER) to form the miRNA duplex which contains the 20–23 nt mature miRNA products [13]. To date, the literature investigating the role of E_2_ in the regulation of miRNAs has primarily focused on regulation of miRNA transcription (i.e., formation of the pri-miRNA). The ability for E_2_ to regulate miRNAs at the level of transcription is conceptually reasonable, and somewhat expected, due to the canonical E_2_ signaling pathways which are mediated primarily by nuclear estrogen receptors (ER)s that act as transcription factors to initiate or inhibit gene expression. Indeed, there have been multiple reports suggesting that E_2_ can have transcriptional effects on miRNAs. For example, E_2_-bound ERβ inhibited the transcription of pri-miR-30a in a breast cancer cell line (MCF7) by binding to two proximal sites near its transcription initiation start site [14]. Notably, E_2_ broadly decreased total miRNA expression in the MCF7 cell line, suggesting that the ERβ-mediated inhibition of transcription could be a shared mechanism to globally downregulate miRNA expression in ER positive cancer cells [15]. Contrary to data from cancer cell models, E_2_ regulation of miRNAs in the brain exhibits distinct molecular patterns; specifically, a global decrease in miRNA expression has not been observed. In fact, our data showed that only a subset of miRNAs were altered by E_2_ treatment in the rat brain, and some were increased while others decreased, raising the potential for E_2_ to selectively target miRNAs in the brain. 

In this study we focused on two of our previously identified E_2_-regulated miRNAs: miR-9-5p and miR-9-3p. These miRNAs are both derived from the same primary transcript (pri-miR-9) and share the same precursor (pre-miR-9), but the mature duplex is unwound to form individual miRNAs with unique mRNA targets [13]. Our data showed that E_2_ treatment increased both miR-9-5p and miR-9-3p in the aged rat hypothalamus, but the pri- and pre-miR-9 levels were unaffected, suggesting that E_2_ regulation of miR-9-5p and miR-9-3p was not at the level of transcription (i.e., pri-) or DROSHA processing (i.e., pre-) [6]. Therefore, the objective of this study was to determine a mechanism for E_2_ regulation of mature miR-9-5p and miR-9-3p and investigate the potential consequences of E_2_ on their ability to effectively repress mRNA translation using polysome profiling. We hypothesized that E_2_ regulates miR-9-5p and miR-9-3p through stabilization of the mature miRNA in an age-dependent manner. We tested this hypothesis using a biochemical approach with neuronal cell lines and hypothalamic tissue isolated from aged female rats. Collectively, we show that E_2_ treatment stabilized miR-9-5p and miR-9-3p in the rat hypothalamus and this stabilization was dependent on age of treatment. Moreover, our data suggest that E_2_ might differentially modulate the ability of miR-9-5p and miR-9-3p to repress the translation of their mRNA targets by shifting their association with polysomes. Together, these data provide a putative mechanistic explanation underlying the “Timing Hypothesis” by highlighting a novel role for E_2_ on mature miRNA degradation.

## 2. Results

### 2.1. E_2_ Stabilized miR-9-5p and miR-9-3p in Neuronal Cells and in the Aged Female Brain

**Figure 1:**
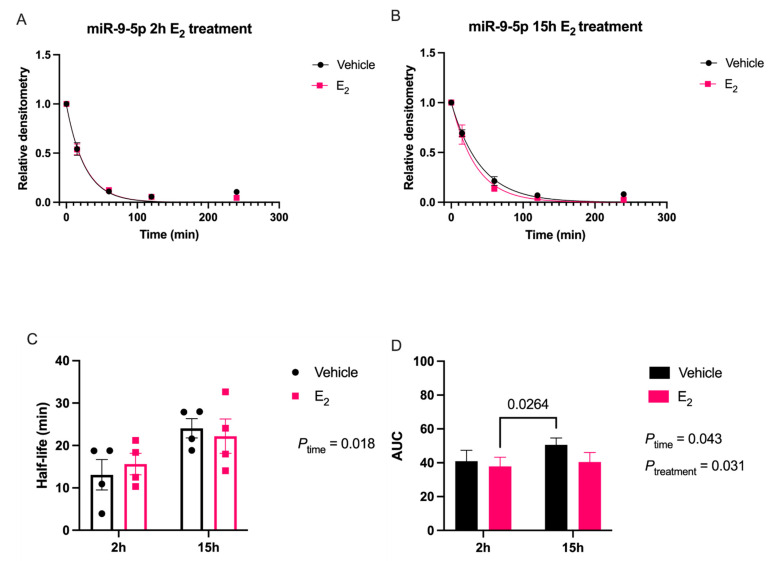
E_2_ treatment increased miR-9-5p stability in hypothalamic-derived neuronal cells. (**A**) Scatterplot of normalized densitometry values analyzed from microradiographs and fit with a one-phase exponential decay function in cells treated with E_2_ for 2 or (**B**) 15 h (black line = vehicle; red line = E_2_; *N* = 4/group). (**C**) Mean half-life and (**D**) area under the curve (AUC) of miR-9-5p calculated from best-fit exponential decay functions. Results are represented as mean ± SEM (*N* = 4/group) and analyzed using two-factor ANOVA for Time and Treatment. Significance was noted when *p* < 0.05. Significant main effects of factors are noted as *P*_subscript_.

We used a previously validated miRNA degradation assay to determine whether E_2_ altered the degradation rate of mature miR-9-5p and miR-9-3p using neuronal cell lines, as described previously [16]. First, we tested the effects of E_2_ using a rat neuronal cell line derived from the paraventricular nucleus (PVN) of the hypothalamus. Our data showed a significant main effect of Time on miR-9-5p half-life in neuronal cells _[F (1, 12) = 7.53; *p* = 0.018]_ (Figure 1A–C). 

**Figure 2:**
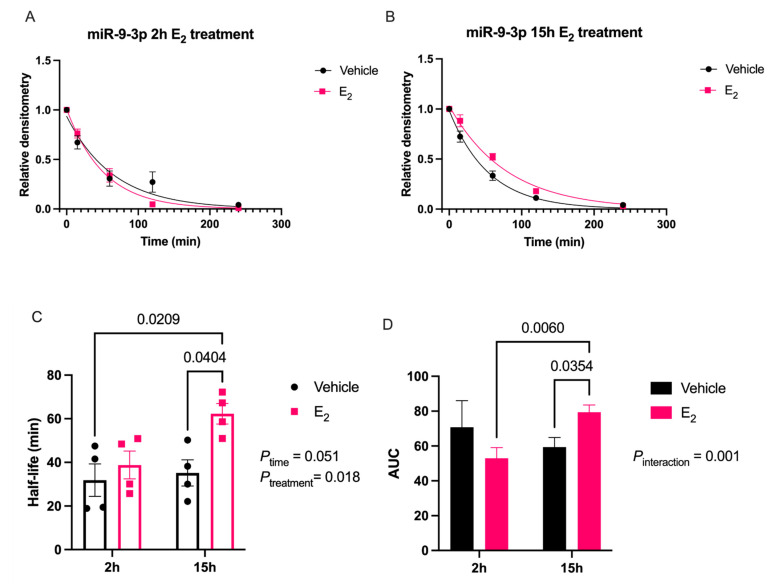
E_2_ treatment increased miR-9-3p stability in hypothalamic-derived neuronal cells. (**A**) Scatterplot of normalized densitometry values analyzed from microradiographs and fit with a one-phase exponential decay function in cells treated with E_2_ for 2 or (**B**) 15 h (black line = vehicle; red line = E_2_; *N* = 4/group). (**C**) Mean half-life and (**D**) area under the curve (AUC) of miR-9-3p calculated from best-fit exponential decay functions. Results are represented as mean ± SEM (*N* = 4/group) and analyzed using two-factor ANOVA for Time and Treatment. Significance was noted when *p* < 0.05. Significant main effects of factors or interactions are noted as *P*_subscript_.

There was also a main effect of Time _[F (1, 12) = 5.1; *p* = 0.043]_ and Treatment _[F (1, 12) = 6.0; *p* = 0.031]_ for miR-9-5p area under the curve (AUC; Figure 1D), but there was no statistically significant interaction between the two factors. A post-hoc analysis of all pair-wise comparisons did not reveal any significant differences between groups for miR-9-5p half-life or AUC. Similarly, E_2_ had robust effects on miR-9-3p stability in neuronal cells (Figure 2).

There was a near significant main effect on miR-9-3p half-life for Time _[F (1, 12) = 4.67; *p* = 0.051]_ and a significant effect of Treatment _[F (1, 12) = 7.53; *p* = 0.018]_, but no significant interaction (Figure 2C). On the other hand, there was a significant interaction _[F (1, 12) = 17.88; *p* = 0.001]_ in miR-9-3p AUC, demonstrating that the effects of E_2_ depended on the length of treatment time (Figure 2D). A post-hoc analysis revealed a significant difference in miR-9-3p half-life between vehicle and E_2_-treated groups after both 2 and 15 h of treatment (Figure 2C). Similarly, there was a significant difference in the AUC between 2 and 15 h of E_2_ treatment, as well as between vehicle and E_2_-treated for 15 h (Figure 2D).

**Figure 3:**
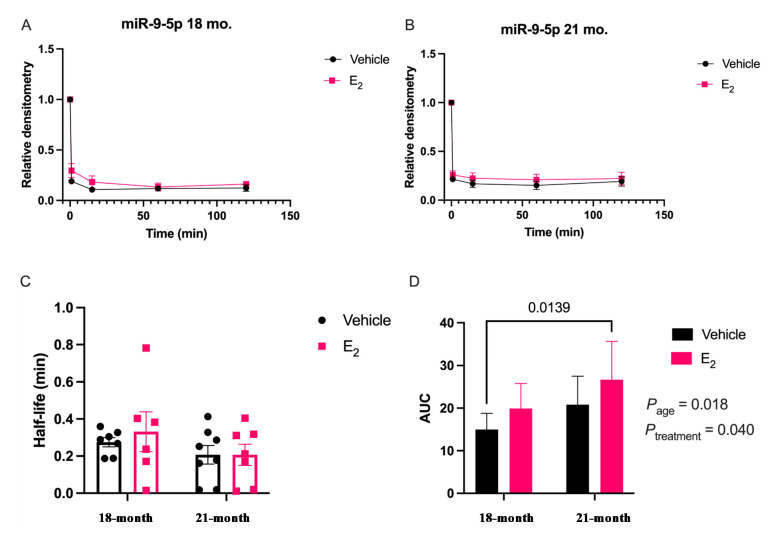
E_2_ treatment increased miR-9-5p stability in the paraventricular nucleus of the hypothalamus in a rat model of menopause. Aged female rats were ovariectomized at 18 months and treated with E_2_ at 1 or 12 weeks following OVX. miRNA degradation assays were performed using brain tissue lysate isolated from the PVN of the hypothalamus. (**A**) Scatterplot of normalized densitometry values were analyzed from microradiographs and fit with a non-linear exponential decay function in 18-month or (**B**) 21-month-old animals (black line = vehicle; red line = E_2_; *N* = 8/group). (**C**) Mean half-life and (**D**) area under the curve (AUC) of miR-9-5p calculated from best-fit exponential decay functions. Results are represented as mean ± SEM (*N* = 8/group) and analyzed using two-factor ANOVA for Age and Treatment. Significance was noted when *p* < 0.05. Significant main effects of factors are noted as *P*_subscript_.

**Figure 4:**
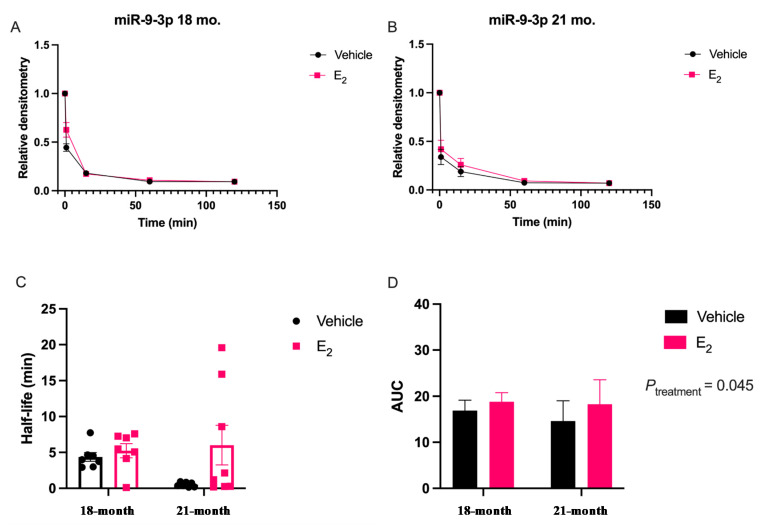
E_2_ treatment increased miR-9-3p stability in the paraventricular nucleus of the hypothalamus in a rat model of menopause. Aged female rats were ovariectomized at 18 months and treated with E_2_ at 1 or 12 weeks following OVX. miRNA degradation assays were performed using brain tissue lysate isolated from the PVN of the hypothalamus. (**A**) Scatterplot of normalized densitometry values analyzed from microradiographs and fit with a non-linear exponential decay function in 18-month or (**B**) 21-month-old animals (black line = vehicle; red line = E_2_; *N* = 8/group). (**C**) Mean half-life and (**D**) area under the curve (AUC) of miR-9-3p calculated from best-fit exponential decay functions. Results are represented as mean ± SEM (*N* = 8/group) and analyzed using two-factor ANOVA for Age and Treatment. Significance was noted when *p* < 0.05. Significant main effects of factors are noted as *P*_subscript_.

Next, we repeated the miRNA degradation assay using PVN tissue samples isolated from a rat model of surgical menopause to further investigate the E_2_-mediated stabilization of miR-9-5p and miR-9-3p in the context of the Timing Hypothesis. Notably, the degradation curves in vivo did not follow a one-phase exponential decay for either miR-9-5p or miR-9-3p (Figure 3 and Figure 4).

Rather, there appeared to be a single rapid cleavage followed by a plateau. However, like our in vitro results, E_2_ treatment in vivo stabilized miR-9-5p and there was a statistically significant main effect on the AUC for Age _[F (1, 24) = 6.44; *p* = 0.018]_ and Treatment _[F (1, 24) = 4.70; *p* = 0.040]_ (Figure 3). However, a post-hoc analysis did not reveal any significant differences between groups and there were no significant differences in half-life, suggesting that E_2_ did not affect the overall rate of degradation kinetics (Figure 3C,D). miR-9-3p had a similar overall degradation profile in the brain and there was a significant main effect of Treatment on the AUC _[F (1, 28) = 4.39; *p* = 0.045]_, but no differences were observed in half-life at either age (Figure 4C,D). Notably, both miR-9-5p and miR-9-3p exhibited rapid degradation kinetics in brain tissue lysate relative to what was observed in the neuronal cells, consistent with previous reports [17,18].

### 2.2. E_2_ Altered miR-9-5p and miR-9-3p Occupancy in Polysome Fractions and the Translation of Downstream mRNA Targets

**Figure 5:**
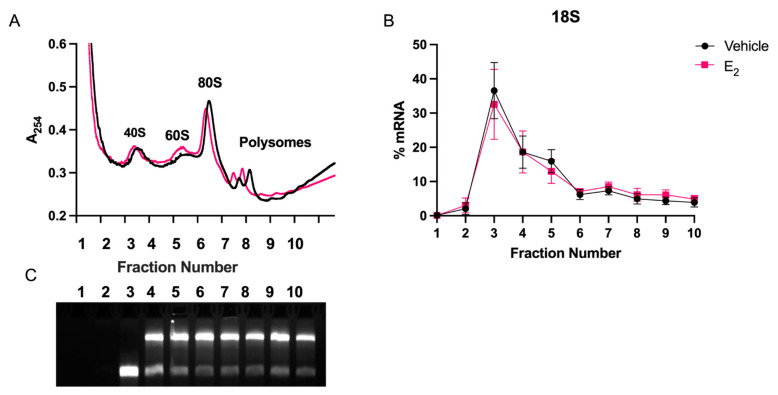
Polysome profiles were not altered following E_2_ treatment in hypothalamic-derived neuronal cells. Cells were treated with E_2_ for 15 h and fractionated using sucrose gradient density sedimentation. (**A**) A254 measurements representing ribosomal or polyribosome subunits and the corresponding fraction numbers for vehicle (black line) or E_2_ treatment (red line). Positions of 40S, 60S and 80S ribosomal subunits and polysomes are indicated. (**B**) 18S polysome profile from cells treated with vehicle or E_2_. (**C**) Representative ethidium bromide RNA gel of 18S and 28S ribosomal subunits recovered in each RNA fraction.

Increased miRNA stability could result in greater efficacy of miRNA-mediated translational repression of mRNA targets. Therefore, we used polysome profiling as a functional proxy for miR-9-5p and miR-9-3p activity to test the effects of E_2_ on miRNA activity in hypothalamic-derived neuronal cells. miRNAs have been shown to copurify with polysomes, reflecting their inhibitory action during active mRNA translation. We first determined that E_2_ treatment did not shift the overall pattern of polysome profiles or significantly alter 18S ribosomal mRNA, demonstrating that E_2_ did not affect global mRNA translation processes (Figure 5).

**Figure 6:**
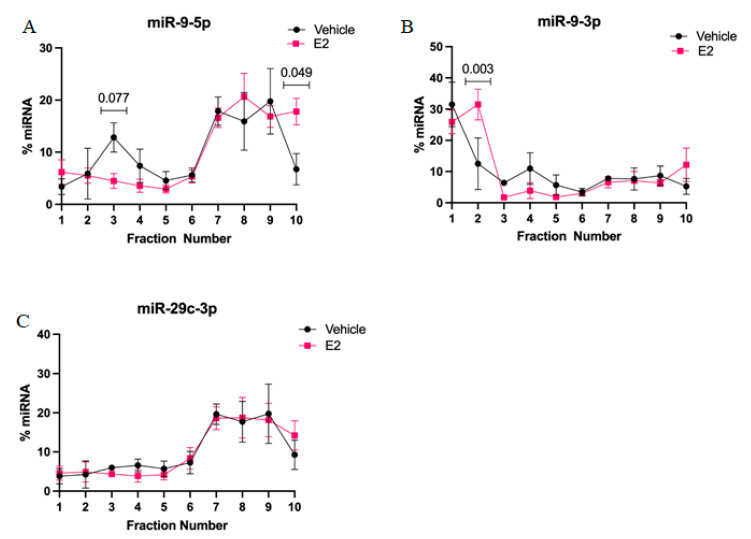
E_2_ altered miR-9-5p and miR-9-3p polysome occupancy in hypothalamic-derived neuronal cells. Cells were treated with E_2_ for 15 h and fractionated using sucrose gradient density sedimentation. Total RNA was isolated from fractions 1–10 and analyzed for expression levels of (**A**) miR-9-5p, (**B**) miR-9-3p, and (**C**) miR-29c-3p. Data were analyzed using unpaired two-sample T-test comparing treatments within each fraction. Data are represented as mean % total mRNA ± SEM (*N* = 3 independent experiments). Significance was noted when *p* < 0.05.

We then measured the occupancy of miR-9-5p and miR-9-3p in the polysome fractions. Our results demonstrated the novel finding that miR-9-5p and miR-9-3p were highly abundant in distinct polysome fractions. Specifically, miR-9-5p was mainly detected in polysome fractions (6–10), while miR-9-3p was most abundant in the 40S ribosomal complex fraction (Figure 6A,B). 

We also observed a significant shift following E_2_ treatment for both miR-9-5p and miR-9-3p; E_2_ treatment shifted miR-9-5p towards a heavier polysome fraction, indicating significantly increased association with more actively translating mRNA transcripts, while E_2_ significantly increased the amount of miR-9-3p in the 40S ribosomal fraction (Figure 6A,B). Moreover, in our polysome factions we measured miR-29c-3p, a microRNA that is not regulated by E_2_ in the hypothalamus. Our data showed that E_2_ treatment did not alter polysome occupancy of miR-29c-3p, indicating that E_2_ regulates specific miRNAs and selectively alters their polysome occupancy.

**Figure 7:**
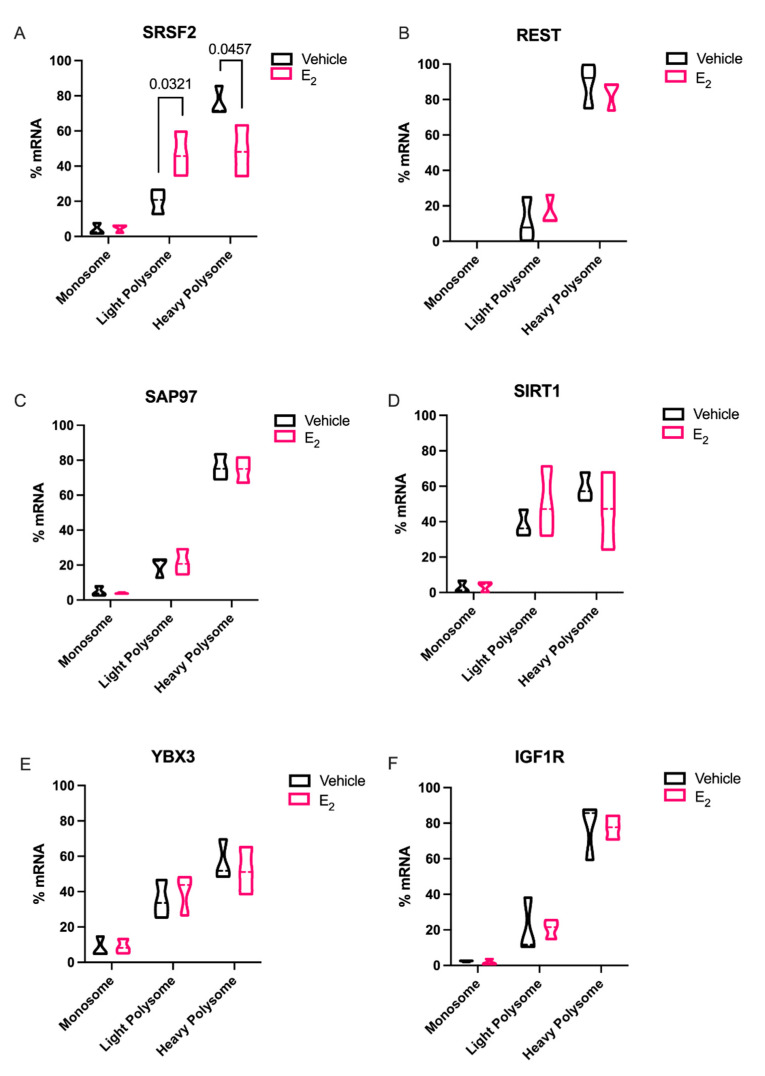
Effects of E_2_ treatment on miR-9-5p and miR-9-3p mRNA gene targets in polysome fractions in hypothalamic-derived neuronal cells. Cells were treated with E_2_ for 15 h and fractionated using sucrose gradient density sedimentation. Total RNA was isolated from pooled from the monosome (fractions 1–4), light polysome (fractions 5–7), and heavy polysome (fractions 8–10) fractions. mRNA expression levels were analyzed as fold change from vehicle treatment using ΔΔCt method. Data are represented as mean % total mRNA ± SEM (N = 3 independent experiments). Significance was noted when *p* < 0.05.

Next, we performed RT-qPCR on pooled polysome fractions for putative miR-9-5p and miR-9-3p target mRNA using the same total RNA isolated for polysome profiling. These experiments tested the downstream consequences of E_2_-mediated miRNA stability by assessing the translational repression of miR-9-5p and miR-9-3p targets: Serine and arginine rich splicing factor 2 (*Srsf2*), RE1 silencing transcription factor (*REST*), Synapse associated protein of 97 kDa (*Sap97*), Sirtuin 1 (*Sirt1*), Y-box binding protein 3 (*YBX3*), and Insulin-like growth factor receptor 1 (*Igf1R*), an E_2_-regulated gene that is a putative, albeit poorly conserved, miR-9-3p target (Table S1) [11,19]. We predicted that E_2_-mediated miR-9-5p and miR-9-3p stability and relocation within polysome fractions would allow for more effective downregulation of their respective mRNA targets. Our results showed that E_2_ treatment significantly reduced the amount of the miR-9-3p mRNA target, *Srsf2*, in the actively translating heavy polysome fraction indicating slow or stalled mRNA translation (Figure 7A). However, other predicted miR-9-5p and miR-9-3p mRNA targets, *Sap97*, *Rest*, *Ybx3, Sirt1, Igf1R*, did not show statistically significant changes in polysome association (Figure 7B–F). 

**Figure 8:**
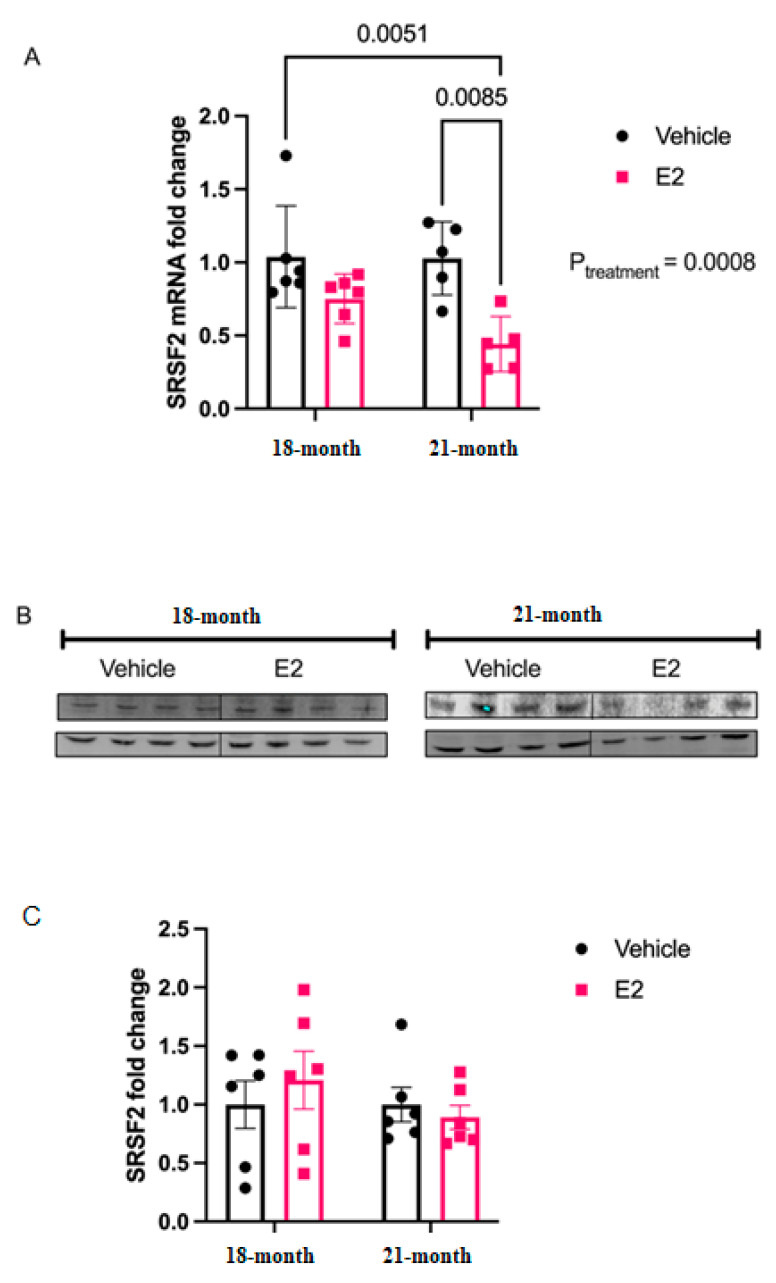
E_2_ treatment decreased *Srsf2* mRNA, but not protein levels, in the paraventricular nucleus of the hypothalamus in a rat model of menopause. (**A**) RT-qPCR results depicting fold change of *Srsf2* mRNA in the PVN of aged female rats with vehicle or E_2_ treatment at 18 or 21 mo. of age. mRNA expression levels were analyzed as fold change from vehicle treatment using ΔΔCt method. (**B**) Representative western blot image of SRSF2 protein and control gene β-actin. (**C**) Quantification of densitometry values from western blots (*N* = 6/group). Data for mRNA and protein quantification were analyzed using two-factor ANOVA for age and treatment. Significance was noted when *p* < 0.05. Significant main effects of factors are noted as *p*_subscript_.

Our polysome profile results demonstrated that E_2_ slowed translation of *Srsf2*. Therefore, we measured *Srsf2* mRNA and protein in our animal model of menopause. Consistent with the Timing Hypothesis, we demonstrated that there was an age-dependent effect of E_2_ on *Srsf2* mRNA expression (Figure 8A). Specifically, there was a main effect of Treatment _[F (1, 24) = 16.36; *p* = 0.0008]_, but not Age, and there was no significant interaction between the factors. A post-hoc analysis revealed that E_2_ treatment at 12-weeks post-OVX (i.e., 21 months old) significantly reduced *Srsf2* mRNA expression in the PVN; however, E_2_ treatment at just one-week post-OVX (18 months old) had no effect (Figure 8A). We also measured protein levels of SRSF2 in the PVN of our animal model. Contrary to what we observed with *Srsf2* mRNA, the protein levels were unchanged with E_2_ treatment in both models (Figure 8B). 

### 2.3. E_2_ did Not Alter the Expression of Critical Components in the miRNA Biogenesis Pathway 

We next quantified the mRNA expression of critical components in the biogenesis pathway using RT-qPCR to determine if E_2_ regulated the transcription of genes important for miRNA biogenesis and/or processing. Consistent with our previous reports, E_2_ did not alter mRNA levels of Argonaute 2 (*Ago2*), 5′-3′exoribonuclease 2 (*Xrn2*), Di-George syndrome critical region 8 (*Dgcr8*), Exportin 5 (*Xpo5*), or *Dicer* in the PVN of our aged female rat model of menopause (Figure S1B–F) [5,6]. Conversely, there was a statistically significant main effect of Treatment on *Drosha* mRNA levels across our two age groups _[F (1, 18) = 6.690; *p* = 0.018]_, but there was no main effect of Age and no interaction (Figure S1A). Moreover, E_2_ treatment did not alter mRNA expression levels of *Ago2, Xrn2, Dgcr8 Xpo5*, *Drosha*, or *Dicer* in our hypothalamic neuronal IVB cells (data not shown). 

## 3. Discussion

The molecular mechanisms underlying the disparate effects of hormone replacement therapy as postulated by the Timing Hypothesis are still being explored. In the present study, we describe the novel finding that E_2_ treatment can regulate mature miRNAs in an age-dependent manner independent of its effects on the pri- and precursor forms of miRNA. The predominant view of how E_2_ regulates miRNAs tends to focus on E_2_-induced changes in miRNA transcription, as mediated by the genomic actions of estrogen receptors (ER)s. Here, we used a biochemical approach to reveal that E_2_ treatment can post-transcriptionally regulate the stability of mature miRNAs in both hypothalamic-derived neuronal cell lines and in the paraventricular nucleus (PVN), a hypothalamic subregion, of aged female rats. Specifically, E_2_ stabilized both strands of the miR-9 duplex (-5p and -3p), which collectively have been shown to be important for driving not only neuronal differentiation, but also for regulating synaptic plasticity in post-mitotic neurons. We also demonstrated the ability of E_2_ to affect mature miRNA activity using polysome profiling, revealing a novel mechanism of miRNA regulation that could contribute to the molecular mechanisms underlying the Timing Hypothesis.

Our data provide evidence that the E_2_-mediated regulation of miR-9-5p and miR-9-3p is executed at the mature level and not at the level of transcription in the aged brain. These results are novel, as other reports have shown that E_2_ regulates various aspects of miRNA biogenesis in other tissue types. For instance, ERs have been shown to bind directly to the promotors of miRNA genes to regulate the transcription of their primary miRNAs [14,20]. Indirect regulation of miRNA transcription has also been reported whereby steroid signaling mechanisms have been shown to recruit other transcription factors to miRNA promoter sites; specifically, c-MYC was recruited to the promotor site of miR-17-92 [21]. In general, E_2_ treatment has been associated with the transcriptional repression of miRNAs, especially when the precursor strand harbors a G-rich terminal loop [15,22]. However, it is important to consider that most of these studies were performed using reproductive tissues, and E_2_ regulation of miRNAs in the central nervous system is notably distinct from peripheral tissues. While E_2_ treatment upregulated levels of mature miR-9-5p and miR-9-3p in the rat hypothalamus, the primary and precursor levels remained unchanged [6], suggesting that this upregulation was not mediated by an increase in transcription. 

Moreover, we generally observed no effect of E_2_ treatment to the miRNA processing machinery in both cell lines and in the PVN of aged female rats. One exception was a significant reduction in *Drosha* mRNA levels in our aged animals. These results from brain tissue contrast with the well-characterized effects of E_2_ on the various aspects of miRNA processing in reproductive tissues. For instance, E_2_ and progesterone signaling concomitantly increased *Xpo5* mRNA expression in mouse uterine tissue, and *Dicer* expression was observed to be increased with E_2_ treatment in MCF7 cells [23,24]. Furthermore, E_2_ positive cell lines had increased *Dicer* and decreased *Ago2* expression compared to ER negative cell lines [25]. In our system, by contrast, we did not observe broad E_2_-mediated changes to the miRNA processing machinery, suggesting that global miRNA processing was not altered by E_2_ treatment. Therefore, E_2_ regulation of miR-9, at least in the aged rat hypothalamus, likely exerts its actions directly at the level of mature miR-9 specific duplex strands.

The subcellular localization of mature miRNAs in the aged rat brain has not been well studied and, to our knowledge, this is the first report showing that E_2_ shifts specific miRNA polysome occupancy. Under cellular stress, active translation shifts towards the formation of processing bodies or stress granules and mRNA translation is stalled [26,27]. On the other hand, miRNA association with cytosolic polysomes is indicative of active translational repression. Therefore, we hypothesized that E_2_-mediated stabilization of miRNAs could subsequently alter the activity and cellular location of miRNA in actively translating polysomes. First, we found that miR-9-5p and miR-9-3p display very different polysome profiles, which likely reflects their guide and passenger strand status, respectively. While miR-9-5p primarily occupies the heavier polysome fractions (~60% total miR-9-5p), miR-9-3p occupies the lightest polysome fractions (~40% total miR-9-3p). Treatment with E_2_ showed a trend, but was not statistically significant, towards decreasing miR-9-5p in the 40S ribosomal complex (i.e., fraction 3), which may partly account for its significantly increased shift in the heavier polysome fractions (i.e., fraction 10). This indicates that E_2_ increased the ability of miR-9-5p to translationally repress its target mRNAs. In contrast, E_2_ significantly shifted miR-9-3p towards the 40S ribosomal subunit (i.e., fraction 2), indicating a role for translational stalling. Moreover, previous studies demonstrated that the 40S ribosome is a marker of stress granules, a cellular compartment that functions to stall translation of mRNAs under stress conditions [27,28]. 

Elongating the half-life of these rapidly degraded miRNAs, as demonstrated with E_2_ treatment, would theoretically afford it more time to repress their mRNA targets. Therefore, we investigated the mRNA targets of both miR-9-5p and miR-9-3p with the expectation that miR-9-5p and/or miR-9-3p targets would be significantly downregulated with E_2_ treatment in polysome fractions. Indeed, *Srsf2*, a predicted target of miR-9-3p was translationally stalled with E_2_ treatment. SRSF2 is a ubiquitous RNA binding protein and is part of the core family of proteins in the spliceosome. Notably, SRSF2 is increased during physiological stress and participates in the alternative splicing of acetylcholinesterase thereby altering cholinergic synaptic transmission in the brain [29,30]. Our finding that E_2_ can inhibit translation of SRSF2 in the aging brain points to a potential mechanism for the protective effects of E_2_ on age-related cellular stress. 

In sharp contrast to our data for *Srsf2* mRNA in polysomes, other putative miR-9-5p and miR-9-3p mRNA targets that we tested were not altered, despite the fact that REST, SAP97, SIRT1, and YBX3 have all been experimentally validated and are highly conserved targets of these miRNAs. These data underscore the importance of considering other cellular factors that might contribute to their translational regulation. For instance, prediction databases such as miRDB highlight several additional miRNAs that could target these same genes, raising the possibility that several miRNAs could work in concert with miR-9-5p and miR-9-3p to achieve full translational repression of these targets [31]. Alternatively, a limitation of our polysome data is that we typically obtain low overall yields of total RNA from individual fractions. Therefore, it was necessary to pool several fractions for the measurements of mRNA, which might have masked differences that might have been otherwise observed in a single fraction. Previous studies in breast cancer cell lines found that E_2_ was able to downregulate REST in polysomes more than total RNA [32]. While a direct relationship of E_2_-mediated miRNA repression of target mRNAs was not established, our data is consistent with the aforementioned study. Our data herein showed that there was a clear trend for E_2_ to decrease levels in the heavy polysome fractions, while increasing it in the light polysome fractions, when comparing the statistical median for REST, SIRT1, and IGF1R, similar to what we observed with SRSF2. 

Estrogens initiate cellular signaling processes via their interaction with nuclear receptors: ERα and ERβ [33]. The stabilizing effect of E_2_ that was observed for miR-9-5p and miR-9-3p in our cell and tissue models was likely mediated primarily by the actions of ERβ. In the hypothalamic-derived IVB cell lines, ERβ is dominantly expressed, though ERβ expression can still be observed at very low levels [34,35]. Additionally, our previous studies showed that an ERβ-, but not ERα-specific agonist, diarylpropionitrile: DPN, significantly increased the expression of miR-9-3p in the rat hypothalamus [6]. Furthermore, ERβ has recently been shown to indirectly interact with Argonaute 2 (*Ago2*) in breast cancer cell lines, suggesting a role for ERβ in RISC loading [36]. Since miRNA loading to *Ago2* has been positively correlated with stabilization of the miRNA [37], it remains an intriguing possibility that ERβ is facilitating the loading of miR-9-3p to the RNA-induced silencing complex (RISC). Furthermore, distinct miRNA profiles were observed between ERβ positive and negative breast cancer cell lines [14], consistent with our previous results in various brain regions. Taken together, these data provide strong evidence that ERβ signaling is critical in the regulation of a specific subset of miRNAs in multiple tissues. 

Our data are consistent with previous reports describing rapid miRNA degradation in the central nervous system [17,18,38], which has been linked to neuronal activity. Here, we demonstrated that miR-9-5p and miR-9-3p degrade on a seconds-to-minutes time scale in hypothalamic-derived neuronal cell lines and in the PVN of aged female rats. Of note, miR-9-5p and miR-9-3p degradation in the neuronal cells exhibited a clear one-phase exponential decay that was not observed in the brain tissue, which instead appeared to have a single-phase decay followed by a plateau. These results could be reflective of the homogenous vs. heterogenous cell types in the brain tissue sample, which is a limitation of this technique. For example, it is possible that miRNA degradation factors differ in neurons compared to glial cells, but to our knowledge these specific factors have not yet been discovered. It is also important to consider that the half-lives derived from this study are not necessarily reflective of endogenous miRNA half-lives, as the lysis process required for the degradation assay disrupts the subcellular. organization of its molecular constituents. This could potentially introduce artificial interactions with degradation factors that would not occur in an endogenous setting. Despite these limitations, it is evident from the obtained biochemical data that E_2_ treatment is altering the cellular milieu (and perhaps degradation factors) in such a way that it is protective of miR-9-5p and miR-9-3p degradation in a time and age-dependent manner. However, delineating how E_2_ treatment impacts the specific kinetics and mechanisms which regulate miRNA stability require further investigation, as previous research has indicated that decay rates influence biological processes and constitute potential drug targets [39].

The molecular switch underlying the time-dependent effects of E_2_ is likely represented by a convergence of multiple signaling pathways. Here, we identify another potential molecular component to this switch in E_2_ action, namely via stabilization of mature miR-9-5p and miR-9-3p, which is observed dependent on age. Due to the importance of these miRNAs in governing normal neuronal function in the adult brain [7,11,40], the results reported herein allow for new avenues of research in understanding how differential miRNA stabilization and activity can determine the efficacy of hormone replacement therapy, particularly in the central nervous system of postmenopausal women.

## 4. Materials and Methods

### 4.1. Animals

Animal procedures were approved by the Institutional Animal Care and Use Committee (IACUC) at Loyola University Chicago (#2009018). All necessary measures were taken to minimize the pain and suffering of animals subject to the Exp. procedures. 18-month-old Fischer 344 rats were obtained from the National Institutes of Aging (NIA) colony at Charles River Laboratories. Rats were pair-housed upon arrival and allowed to acclimate to their environment for one week prior to further experimentation. Rats were supplied with standard rat chow and tap water ad libitum and were kept on a 12/12 h. light/dark cycle with Zeitgeber time (ZT) 0 at 7 A.M. Animals were ovariectomized (OVX) at 18 months of age after the acclimation period and then left undisturbed for 1 or 12 weeks following OVX, therefore animals were 18 months and 21 months, respectively, at the time of euthanasia. These animal procedures were followed as outlined by Rao and colleagues [5]. After the designated time intervals post-OVX, the animals were given a subcutaneous injection of either safflower oil or 2.5 μg/kg 17β-estradiol E_2_ dissolved in safflower oil once/day for 3 consecutive days. This dose has been previously reported to achieve circulating E_2_ concentrations within the physiological range for postmenopausal women receiving HRT (17–75 pg/mL) [6,41,42]). Animals were euthanized 24-h after the final injection.

### 4.2. Ovariectomy

Animals were deeply anesthetized with vaporized isoflurane and bilaterally ovariectomized (OVX), as described previously [6]. Briefly, the ovary and distal end of the uterine horn were excised from the body cavity after the uterine horn was clamped with a hemostat and ligated proximal to the clamp. Animals were singly housed and provided with acetaminophen analgesic in tap water for 3 days following the procedure. After 3 days of analgesia, the animals were pair-housed with their previous cage mate and were undisturbed for the duration of the experiment.

### 4.3. Tissue Processing

The animals were deeply anesthetized using vaporized isofluorane and euthanized by rapid decapitation. Brains were rapidly dissected, flash-frozen in 2-methylbutane at −30 °C, and then sectioned coronally at 200 μm on a freezing microtome (Leica Biosystems, Lincolnshire, IL, USA). The paraventricular nucleus (PVN) was microdissected using a 0.75-mm Palkovit’s brain punch tool (Stoelting, Inc., Wood Dale, IL, USA) at −1.49 to −2.12 relative to bregma, as defined by *The Rat Brain in Stereotaxic Coordinates* [43]. Frozen PVN microdissections were transferred to a microcentrifuge tube and stored at −80 °C.

### 4.4. Cell Culture

For in vitro studies, we used a neuronal cell line derived from the paraventricular nucleus (PVN) of the rat hypothalamus (IVB cells, provided by John Kaskow, University of Cincinnati, Cincinnati, OH, USA). Cells were maintained in normal growth media (DMEM media containing glucose, L-glutamine, sodium pyruvate, and 10% fetal bovine serum (FBS) and grown to 60–70% confluency prior to experiments. For all Exp. conditions, cells were maintained in media containing 10% charcoal/dextran stripped FBS (substituted for regular FBS) for 48 h to eliminate all endogenous sources of hormones from the FBS. After 48 h, cells were treated with 100 nM E_2_ or an equivalent volume of media containing 0.001% ethanol (vehicle) for 2 or 15 h before lysis.

### 4.5. RNA Isolation and cDNA Synthesis

Total RNA was isolated from IVB cells and PVN tissue microdissections using the Zymogen DirectZol kit (Zymo Research, Irvine, CA, USA). Total RNA (1.0 μg) was reversed transcribed using the Norgen miRNA cDNA Synthesis kit (Thorold, ON, Canada) or Invitrogen Superscript IV mRNA synthesis kit for mRNA Cdna (Waltham, MA, USA), according to manufacturer instructions. 

### 4.6. RT-qPCR

All mRNA transcripts (i.e., miRNA biogenesis components and targets) were quantified by RT-qPCR using the procedures and primers previously described [6]. Each individual biological sample was assayed as triplicate replicates within an assay. We used 18S rRNA as a housekeeping gene that has previously been verified as unaffected by E_2_ treatment [44]. Transcripts were quantified relative to vehicle-treated control using the ΔΔCt method [45]. The following conditions were used for the thermocycler: (1) 95 °C for 10 min, (2) 95 °C for 15 s, (3) 59 °C for 20 s, and (4) 72 °C for 12s in addition to melting curve analysis. 

### 4.7. miRNA Degradation Assay

The miRNA degradation assay was adapted for tissue and whole cell lysates according to methods originally described by [46], and then modified by Kim and colleagues [18]. Briefly, oligonucleotide constructs were synthesized with the exact nucleotide sequence of the mature transcript for miR-9-5p and miR-9-3p, [UCUUUGGUUAUCUAGCUGUAUGA] and [AUAAAGCUAGAUAACCGAAAGU], respectively (Integrated DNA Technologies, Coralville, IA, USA). These single stranded oligonucleotide sequences were then radiolabeled on the 5′ end using [γ32P] ATP (3000 Ci/mmol; PerkinElmer, Waltham, MA, USA); at a ratio of 10 fmols: 20 μg for the radiolabeled RNA and protein from either the IVB cell lysate or brain tissue lysate, respectively. Incubation of the radiolabeled miRNA oligonucleotides with the lysate was terminated at five different time points by boiling at 95 °C for 2 min following the addition of 2 × RNA Loading Dye (New England Biolabs, Ipswich, MA, USA). The resulting mixture was then resolved on an 8% urea gel by electrophoresis. Finally, the gel was visualized by phosphoimaging (Typhoon GE Healthcare, Chicago, IL, USA) to detect levels of the radiolabeled miRNA at the various time points. Gel bands were quantified using densitometry analyses with ImageJ (RRID:SCR_003070) v5.2 software and averaged densitometry values from multiple replicates were plotted on a scatterplot using OriginLab software (v8.5.1, Northampton, MA, USA). Prism software (v9.1.0, San Diego, CA, USA) was used to determine degradation kinetics using a non-linear regression, one-phase decay, half-life calculated from the best fit exponential decay function; specifically, T_1/2_ = ln2*tau and area under the curve (AUC). Data were analyzed by two-factor ANOVA, followed by Tukey post-hoc for multiple pair-wise comparisons.4.8. Western Blot

Protein lysate from IVB cells and tissue was prepared using a 0.5% NP40 buffer with protease and phosphatase inhibitors (Thermo Fisher Scientific, Skokie, IL, USA). Following lysis procedures, protein concentration was determined using a bicinchoninic acid (BCA) assay according to manufacturer instructions (Thermo Fisher Scientific, Skokie, IL, USA). Total protein (30 μg) was boiled with 4X Laemmli buffer (BioRad, Hercules, CA, USA) at 95 °C for 5 min before electrophoresis on a 10% polyacrylamide gel. Following gel electrophoresis at 120 V for 1 h, proteins were transferred to Immobilon™ PVDF membranes (Millipore, Burlington, MA, USA) at 100 V for 1 h at 4 °C. Membranes were blocked with a 1:1 solution of TBS and Odyssey blocking solution (Li-cor Biosciences, Lincoln, NE, USA) for 1 h at room temperature. Following the blocking procedure, membranes were incubated with the indicated primary antibody overnight with constant agitation at 4 °C. Membranes were then incubated with 1:1000 secondary antibodies, as appropriate (Li-cor Biosciences, Lincoln, NE, USA), for 1 h at room temperature. Protein bands were visualized using the Azure Biosystems imaging system (Dublin, CA, USA). Densitometry values were calculated after subtracting background and relative fold changes were made compared to vehicle controls.

### 4.8. Polysome Profiling

Hypothalamic-derived neuronal IVB cells were grown in media containing 10% charcoal-stripped FBS for 48 h, then treated with E_2_ (100 nM) or vehicle (0.001% EtOH) for 15 h, followed by 0.1 mg/mL cycloheximide (Sigma-Aldrich, St. Louis, MO, USA) for 10 min prior to lysis. Cells were lysed in polysome extraction buffer [47] and protein was quantified by BCA to ensure equal loading between each sample. 1.5 mg lysate was loaded onto a 15–45% sucrose gradient. Gradients were centrifuged for 90 min at 19,0000× *g* at 4 °C. Fractions were collected on ice at 1.5 mL/min into 10 tubes per gradient. Absorbance was measured at 254 nm using a Brandel UA-6 Type 11 absorbance detector and data was recorded with Brandel Peak Chart data capture software to record polysome profiles (Gaithersburg, MD, USA). Trizol was immediately added to fractions and stored at −80 °C until RNA isolation. All RNA samples were collected using column purification (Zymo, Irvine, CA, USA). 200 ng of total RNA per fraction was used for miRNA or mRNA cDNA synthesis, as described above. RT-qPCR analysis of miRNAs in each fraction was normalized to 18S mRNA, which did not show shifts in distribution patterns with treatment. Each assay was repeated in 3–4 independent experiments, with 3 technical replicates per experiment.

### 4.9. Statistics

Statistical analyses were performed using Prism software (v9.1.0, San Diego, CA, USA). Data were analyzed by two-factor ANOVA followed by Tukey post-hoc test for multiple pair-wise comparisons unless otherwise noted in figure captions. Data are displayed as mean ± SEM and statistical significance was determined at *p* < 0.05.

## Data Availability

Data is contained within the article or supplementary material.

## Supplementary Materials

The following are available online at https://www.mdpi.com/article/10.3390/ncrna7030053/s1, Figure S1: Effects of E2 treatment on mRNA levels of miRNA processing machinery in the paraventricular nucleus of the hypothalamus (PVN) in a rat model of menopause, Table S1: Table of experimentally validated targets of miR-9-5p and miR-9-3p.
