# Supplementary material for: 17β-Estradiol Regulates miR-9-5p and miR-9-3p Stability and Function in the Aged Female Rat Brain
Source: Noncoding RNA. 2021 Aug 30;7(3):53. doi: 10.3390/ncrna7030053 (PMC8482090; doi:10.3390/ncrna7030053)
Supplement: Supplementary file 1 [file ncrna-07-00053-s001.zip › ncrna-1337347-supplementary.pdf]

SUPPLEMENTAL DATA

Table S1. Table of experimentally validated mRNA targets of miR-9-5p and miR-9-3p. Validated targets were identified by miRTarBase. Only targets that have been validated by reporter assay, weatern blot, or RT-qPCR are represented.

miRNA	Validated Targets
miR-9-5p	CCND1, FOXO3, ID2, SOCS5, TGFB1, SIRT1, SRF, MMP13, REST, CDH1, STMN1, POU2F2, BCL6, ETS1, RAB34, BACE1, FOXG1, PRDM1, YBX3 HER9, NFKB1, RUNX1, HER5, FGFR1a, CNPY1, FGF8a, NR2E1, ONECUT2, FOXP1, CDX1, HES1, ZFP521, AP3B1, CCNG1
miR-9-3p	RCOR1, SAP97, ITGB1, GNAI1, SRSF2

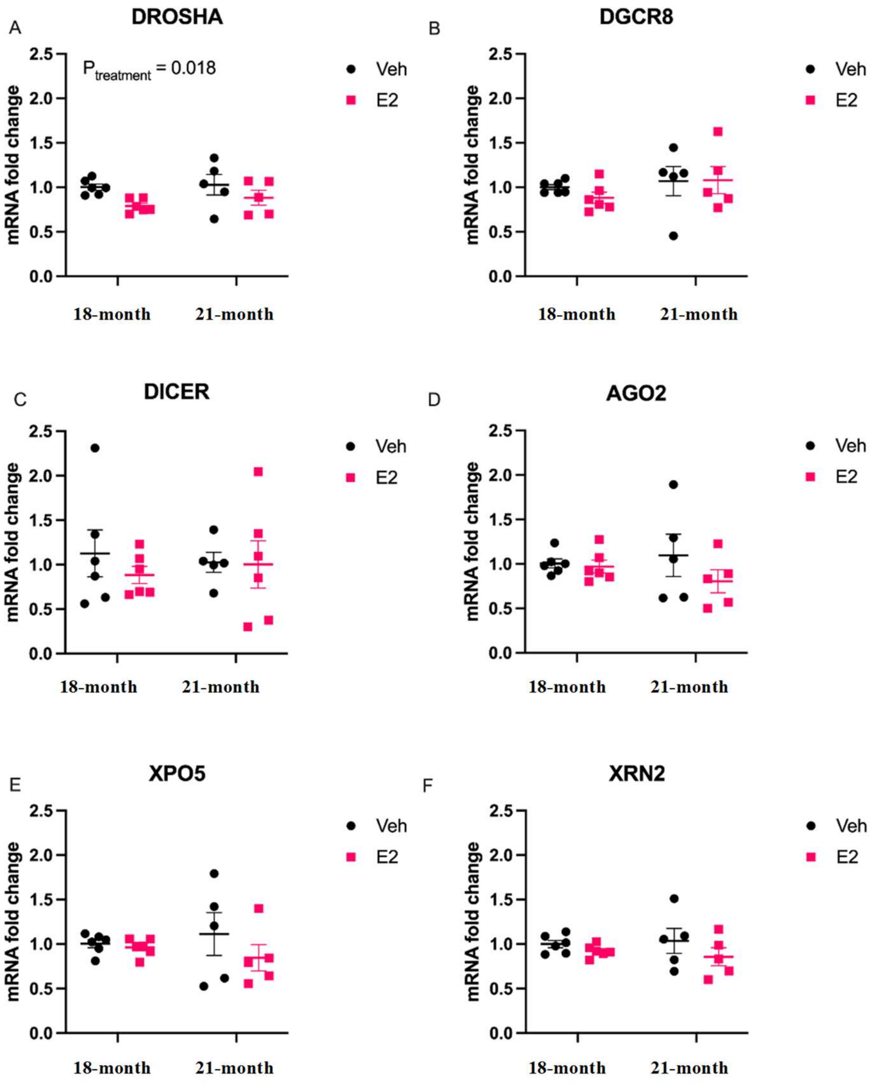

Figure S1. Effects of E_2 treatment on mRNA levels of miRNA processing machinery in the paraventricular nucleus of the hypothalamus (PVN) in a rat model of menopause. RT-qPCR results depicting relative fold change of (**A**) *Drosha*, (**B**) *Dgcr8*, (**C**) *Dicer*, (**D**) *Ago2*, (**E**) *Xpo5*, and (**F**) *Xrn2* mRNA in the PVN of aged female rats treated with vehicle or E_2 treatment at 1- or 12-weeks following OVX, 18 mo. and 21 mo. old respectively. Results are represented as mean ± SEM (N = 6/group) and analyzed using two-factor ANOVA for Age and Treatment. Significance was noted when $p<0.05$. Significant main effects of factors are noted as $P_{subscript}$.
